# Acute Ammonia Causes Pathogenic Dysbiosis of Shrimp Gut Biofilms

**DOI:** 10.3390/ijms25052614

**Published:** 2024-02-23

**Authors:** Ning Gao, Yi Shu, Yongming Wang, Meng Sun, Zhongcheng Wei, Chenxi Song, Weipeng Zhang, Yue Sun, Xiaoli Hu, Zhenmin Bao, Wei Ding

**Affiliations:** 1 Key Laboratory of Tropical Aquatic Germplasm of Hainan Province, Sanya Oceanographic Institution, Ocean University of China, Sanya 572000, China; ninggao1005@163.com (N.G.); 15254135903@163.com (Y.S.); wymsyp12@163.com (Y.W.); sunyue@ouc.edu.cn (Y.S.); 2 Southern Marine Science and Engineer Guangdong Laboratory, Guangzhou 511458, China; 3 MOE Key Laboratory of Marine Genetics and Breeding, Ocean University of China, Qingdao 266003, China; weizhongcheng@stu.ouc.edu.cn (Z.W.); 13397712090@163.com (C.S.);; 4 Institute of Evolution & Marine Biodiversity, Ocean University of China, Qingdao 266003, China; sunmeng950103@163.com (M.S.); zhangweipeng@ouc.edu.cn (W.Z.)

**Keywords:** ammonia exposure, *Litopenaeus vannamei*, gut microbiota, *Vibrio*

## Abstract

Acute ammonia exposure has detrimental effects on shrimp, but the underlying mechanisms remain to be fully explored. In the present study, we investigated the impact of acute ammonia exposure on the gut microbiota of the white shrimp *Litopenaeus vannamei* and its association with shrimp mortality. Exposure to a lethal concentration of ammonia for 48 h resulted in increased mortality in *L. vannamei*, with severe damage to the hepatopancreas. Ammonia exposure led to a significant decrease in gut microbial diversity, along with the loss of beneficial bacterial taxa and the proliferation of pathogenic *Vibrio* strains. A phenotypic analysis revealed a transition from the dominance of aerobic to facultative anaerobic strains due to ammonia exposure. A functional analysis revealed that ammonia exposure led to an enrichment of genes related to biofilm formation, host colonization, and virulence pathogenicity. A species-level analysis and experiments suggest the key role of a *Vibrio harveyi* strain in causing shrimp disease and specificity under distinct environments. These findings provide new information on the mechanism of shrimp disease under environmental changes.

## 1. Introduction

The white shrimp *Litopenaeus vannamei* is a cultivated worldwide species due to its adaptation to a broad range of salinity [1]. In recent decades, shrimp farming in tropical and subtropical coastal areas has experienced rapid expansion, becoming an increasingly important sector of marine aquaculture [2]. As of 2020, China’s shrimp aquaculture production has reached 2.6 million tons, exhibiting a 5.2% average annual growth rate [3]. However, abominable aquaculture environments as a result of high nitrogenous waste concentrations substantially impact shrimp production, resulting in significant economic losses worldwide [4].

The final product of protein catabolism in crustaceans, ammonia, accounts for 60–70% of nitrogen excretion, with relatively minor amounts of amino acids, urea, and uric acid [5]. It also accumulates in the culture system due to a variety of conditions, such as insufficient water flow, high stocking density, and excessive feeding [6]. Ammonia buildup endangers shrimp health and survival, as it harms the gills, impairs immunological function, and limits the ability of hemolymphs to carry oxygen while increasing tissue oxygen use [7,8,9]. Ammonia can also cause oxidative stress in shrimp, including a decline in superoxide dismutase (SOD) activity and an improvement in malonyl dialdehyde (MDA) levels [10,11]. In addition, the mortality rate of post-larvae increases with rising ammonia concentrations [12]. Consistently, a transcriptomic analysis of the hepatopancreas of *M. japonicus* exposed to acute ammonia revealed that antioxidants, apoptosis pathways, tissue injuries, and oxidative stress were markedly activated by ammonia [13,14].

While the above-mentioned studies have provided insights into the impact of ammonia on shrimp, very little attention has been paid to the effect of ammonia on the shrimp gut microbiota. The gut microbiota, as a kind of microbial biofilm, has been known to play important roles in dealing with environmental stimuli and in helping the host adapt to a variety of environments [15,16,17]. In one study, exposure to ammonia resulted in taxonomic abundance and composition variations in the gut microbiota of *L. vannamei* [18]. *Mesorhizobium* (20.66%), *Halomonas* (17.94%), and *Sphingomonas* (12.26%) were prevalent in the gut microbiota of *L. vannamei* after exposure to ammonia (20 mg/L, less than the 96 h lethal concentration) for 96 h [18]. However, the correlation between death due to ammonia and a change in gut microbial composition remains elusive.

In the present study, *L. vannamei* was exposed to the 48 h lethal concentration (71.68 mg/L) of ammonia. The shrimps were categorized into three groups: the control group (without ammonia exposure), the diseased group (after ammonia exposure), and the dead group (after ammonia exposure). Additionally, the change in the gut microbial diversity and composition after exposure to ammonia were comparatively examined. Then, functional prediction and infection experiments using isolated strains were performed for a mechanistic understanding of ammonia-caused death of shrimps.

## 2. Results

### 2.1. Experimental Design and the Impact of Ammonia Exposure on Shrimp Hepatopancreas

**Figure 1:**
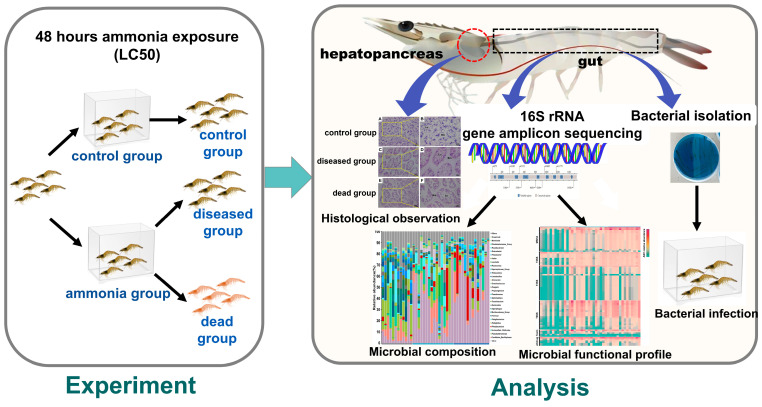
A schematic diagram of the experimental design.

**Figure 2:**
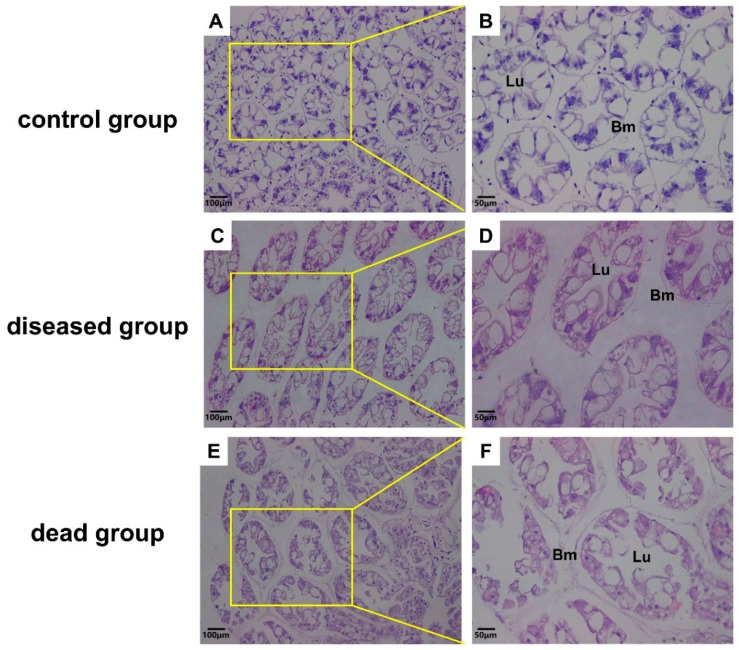
Histopathological changes in hepatopancreas of *Litopenaeus vannamei* in different groups. Lu: lumen, Bm: basement membrane. (**A**,**C**,**E**) Magnification: 20×. Scale bar: 100 μm. (**B**,**D**,**F**) Magnification: 40×. Scale bar: 50 μm.

After the shrimps were exposed to a series of concentrations of ammonia, the cumulative mortality rate of *L. vannamei* gradually increased with the increase in the ammonia concentration (Figure S1A). The 48 h LC50 was calculated using the formula lgLC50 = 1/2(X_i_ + X_i+1_) (P_i+1_ − P_i_) (Figure S1B), according to which 71.68 mg/L of ammonia was determined as the 48 h LC50 and was used for the following exposure experiment, as shown in Figure 1. Prior to the treatment, the shrimps (*L. vannamei*) were categorized into two groups, with 20 shrimps as the control group and 50 shrimps exposed to 71.68 mg/L of ammonia (referred to as the ammonia group, Figure 1). After 48 h, the ammonia group was further categorized into the diseased group (*n* = 27) and the dead group (*n* = 23), according to the state of the shrimps (Figure 1). Among them, 5 shrimps were used for hepatopancreas histopathology, 17 shrimp guts were subjected to DNA sequencing, and the remaining shrimp guts were used for strain isolation. Due to sequencing failure, one sample from the diseased group and three samples from the dead group did not yield sufficient data. Thus, 16 samples in the diseased group and 14 samples in the dead group were included for further analysis. Compared to the control group, shrimp in the ammonia group displayed tissue damage and structural disruption in their hepatopancreas (Figure 2). In the control group, the hepatopancreas exhibited a well-organized glandular structure with evenly sized hepatic tubules and intact basement membranes (Figure 2A,B). However, in the ammonia group, significant damage was noticed in both the diseased and dead shrimps. The hepatopancreatic lumen of the diseased shrimp was expanded, with mild distortion and disorganized hepatic tubules (Figure 2C,D). The dead shrimp exhibited considerably more serious damage, such as epithelial cell rupture, lumen degeneration, loose tubule organization, and cell–cell separation from the basement membrane (Figure 2E,F).

### 2.2. A Decrease in Gut Microbial Diversity on Exposure to Ammonia

To compare gut microbial compositions with and without ammonia exposure, 16S rRNA amplicons were sequenced from 12 shrimps from the control group, 16 shrimps from the ammonia diseased group, and 14 shrimps from the ammonia dead group. A total of 3,281,145 raw reads were generated, with each sample having 71,693 ± 20,745 reads (Table S1). For each sample, 200–620 ASVs were obtained, amounting to a total of 5596 ASVs (Table S1). A rarefaction analysis was performed to verify the sequencing depth (Figure S2).

**Figure 3:**
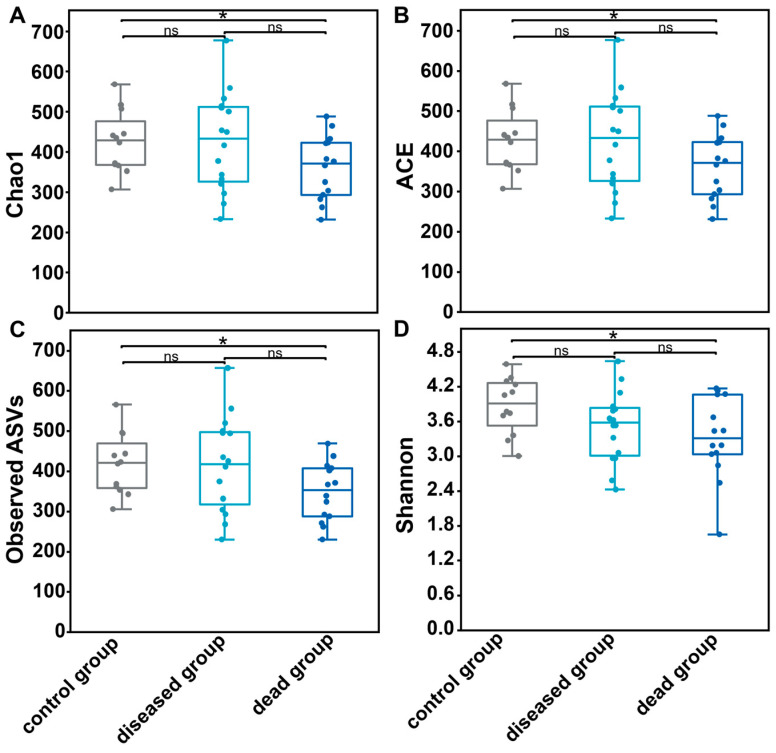
Alpha diversity of the gut microbiota. (**A**) Chao_1 index. (**B**) Abundance-based coverage estimator (ACE) index. (**C**) Observed amplicon sequence variants (ASVs). (**D**) Shannon index. Significant differences among different groups were examined using two-tailed Student’s *t*-test. * *p* < 0.05. ns: non-significant.

The microbial richness was reflected in the Chao_1 index, abundance-based coverage estimator (ACE), and observed ASVs. There was no significant difference between the control and the ammonia diseased group (Figure 3A–C); nonetheless, the median values of these analyses in the ammonia dead group were significantly lower (two-tailed Student’s *t*-test, *p* < 0.05) in comparison with the control or the ammonia diseased group (Figure 3A–C). Meanwhile, the median values of Shannon diversity in the ammonia group were 3.5 and 3.32, respectively, for shrimps in the diseased and dead groups, which were much lower than that in the control group (Shannon value of 3.91) (Figure 3D). These results indicated that acute ammonia exposure of shrimp could decrease the microbial richness in the ammonia group, but only in the dead shrimps. Concurrently, acute ammonia exposure significantly impacted the gut microbial diversity of shrimp in both the diseased and dead groups.

### 2.3. Ammonia Exposure Shifted the Gut Microbial Composition

**Figure 4:**
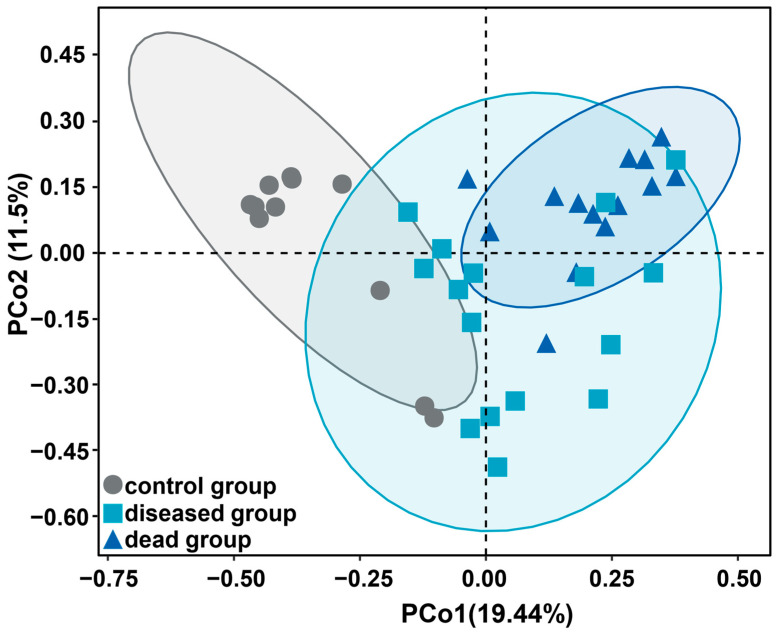
Principal coordinate analysis of taxonomic composition based on Jaccard distance matrix. The samples are color-coordinated to represent different groups.

PCoA based on Jaccard distances was conducted to reveal the taxonomic structural similarity of gut microbiota from different groups. The gut microbiota from the ammonia dead group were significantly different from those in the control group, whereas gut microbiota from the ammonia diseased group overlapped with those from the other two groups (Figure 4), suggesting that the gut microbiota of the dead shrimps was more profoundly impacted than the diseased group.

All ASV representative sequences were taxonomically classified, and the read counts aggregated at the phylum and genus levels. At the phylum level (Proteobacteria were classified down to class level), 37 phyla were identified. Consistent with the PcoA result, there was a clear variation in gut microbial structure between different groups, especially between the control and the ammonia dead groups (Figure S3). Gammaproteobacteria were dominant in all groups and reached up to 6–61% in the control group, 21–88% in the ammonia diseased group, and 47–93% in the ammonia dead group (Figure S3). In the control group, Bacteroidetes (6–54%) was specifically abundant, followed by Alphaproteobacteria (5–34%) (Figure S3). In the ammonia diseased group, Tenericutes (2–35%) and Bacteroidetes (1–53%) were specifically abundant, followed by Alphaproteobacteria (2–23%) (Figure S3).

A total of 854 genera were identified. Overall, all genera were evenly distributed in the control group, with *Halioglobus* (4–26%), *Candidatus*_Bacilloplasma (1–19%), *Pelagibacterium* (0.3–30%), and *Vibrio* (2–37%) as the relatively abundant genera (Figure S4). In contrast, ammonia exposure led to increased proliferation of *Vibrio*, especially in the ammonia dead group and a decrease in certain Roseobaceters, such as *Ruegeria* (Figure S4). Specifically, *Vibrio* accounted for 4–75% of the microbiota in the ammonia diseased group and 23–91% in the ammonia dead group. Notably, *Photobacterium*, which also includes many pathogenic bacteria, was also enriched in the ammonia dead group (Figure S4).

**Figure 5:**
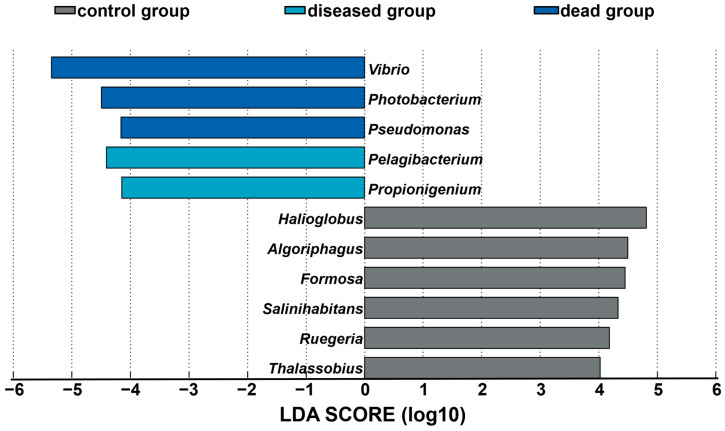
Linear discriminant analysis (LDA) effect size (LefSe) analysis among different groups of gut microbiotas. Significantly different taxa based on effect size (LDA score [log 10] > 4) are shown here. Enriched taxa in the ammonia groups had positive LDA scores while enriched taxa in the control group had negative LDA scores.

The proliferation of pathogenic bacteria after ammonia exposure was further confirmed by an LefSe analysis. According to the LDA score (LDA > 4, *p* < 0.05), eleven significantly changed genera were identified among different groups (Figure 5). Six genera were over-represented in the control group and included *Halioglobus*, *Algoriphagus*, *Formosa*, *Salinihabitans*, *Ruegeria*, and *Thalassobius*. After exposure to ammonia, *Pelagibacterium* and *Propionigenium* showed the highest LDA values in the ammonia diseased group, while *Vibrio*, *Photobacterium*, and *Pseudomonas* were over-represented in the ammonia dead group. A violin plot displayed the distribution and variability of *Vibrio* across different groups (Figure S5). Consistent with the results of the LefSe analysis, two-tailed Student’s *t*-test indicated significantly enhanced relative abundance of *Vibrio* (*p* < 0.05) in the ammonia groups compared to the control group (Figure S5). Moreover, the distribution pattern of *Vibrio* across different samples was squat in the ammonia dead group and rangy in the other two groups (Figure S5). A further statistical analysis using one-way ANOVA on the *Vibrio* ASVs displayed significant variations between the groups (Figure S6). Twelve *Vibrio* ASVs were detected, with most of them being significantly enriched in the ammonia groups, particularly the ammonia dead group. In detail, ASV001 exhibited notable relative abundance in the ammonia dead group and was the most representative ASV after exposure to ammonia (Figure S6).

### 2.4. Phenotypic and Functional Change in the Shrimp Gut Microbiota

**Figure 6:**
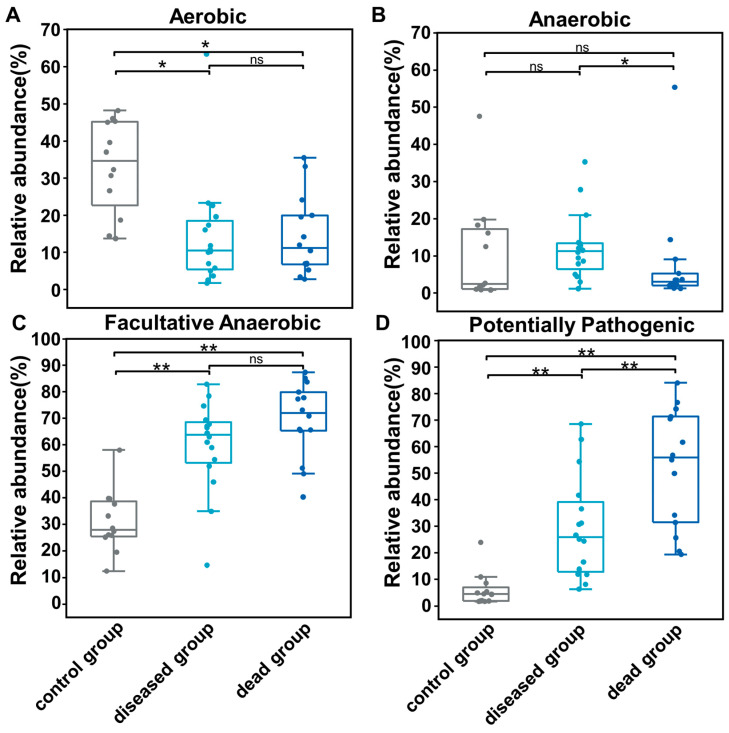
The transition of gut microbial phenotype as calculated using BugBase. Microbial phenotype, including aerobic (**A**), anaerobic (**B**), facultative anaerobic (**C**), and potentially pathogenic (**D**) phenotypes. Two tailed Student’s *t*-test was used here. * *p* < 0.05; ** *p* < 0.01. ns: non-significant.

Phenotypic change caused by ammonia exposure was assessed by a BugBase analysis (Figure 6). Aerobic microbes diminished dramatically after ammonia exposure and anaerobic microbes showed no significant change, whereas facultative anaerobic microbes significantly bloomed (Figure 6). Moreover, potentially pathogenic bacteria were significantly and highly enriched after ammonia exposure, especially in the ammonia dead group (Figure 6).

**Figure 7:**
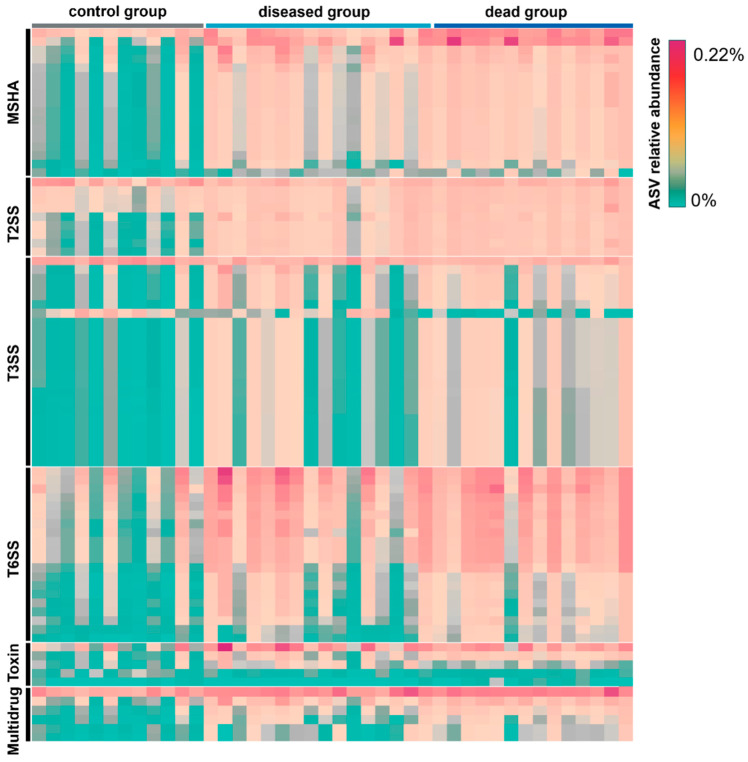
The prediction of pathogenic-related genes in the gut microbial community across different groups. The significantly changed genes across different groups (one-way analysis of variance *p* < 0.05) are shown here. MSHA: mannose-sensitive hemagglutinin; T2SS: type II secretion system; T3SS: type III secretion system; T6SS: type VI secretion system.

Pathogenicity-related metabolic variations across different groups were assessed by predicting the functional dynamics associated with the transition of taxonomic structure after ammonia exposure using PICRUSt2 (Figure 7). In total, 82 representative genes affiliated with the microbial taxa were identified. Among these, genes related to mannose-sensitive hemagglutinin (MSHA), type II secretion system (T2SS), type III secretion system (T3SS), type VI secretion system (T6SS), toxins, and multiple drugs were enriched in the ammonia groups (Figure 7). By contrast, the control group had a lower relative abundance of genes involved in pathogenicity. Genes involved in MSHA and T6SS were specifically enriched in the ammonia dead group more than in the ammonia diseased group (Figure 7).

### 2.5. Pathogenicity of Vibrio Strains Isolated from Gut Microbiota of the Ammonia-Treated Shrimps

We next experimentally confirmed the pathogenicity of *Vibrio* strains. Comparing 16S rRNA gene sequences revealed that strain *Vibrio* SXD57 had 100% sequence similarity with ASV001, which was significantly enriched in the ammonia dead group. A phylogenetic tree indicated that SXD57 had the closest relationship with *Vibrio harveyi* 2–25, with a bootstrap value of 86 (Figure S7). Thus, this strain was named as *V. harveyi* SXD57.

**Figure 8:**
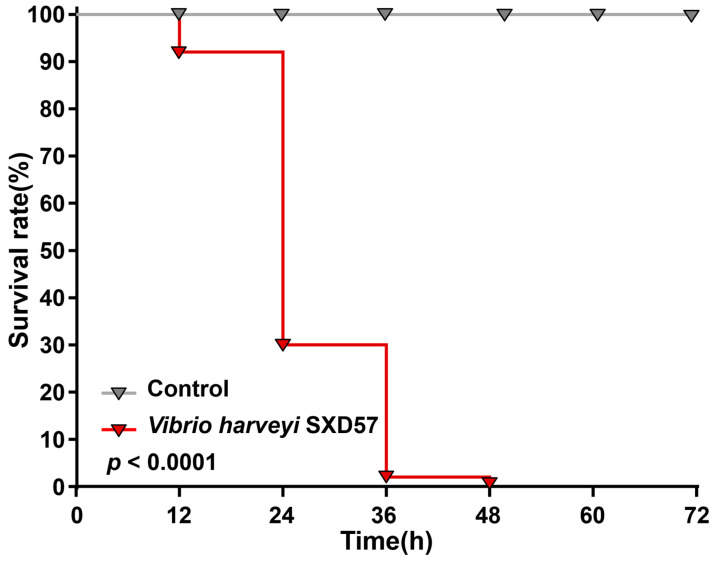
The 72 h shrimp survival rate after *Vibrio harveyi* infection. Gehan–Breslow–Wilcoxon test was used to examine the significance of the difference between the treatment and the control group.

Next, the SXD57 culture was used to infect healthy shrimps and survival rates with 72 h were recorded with an interval of 12 h. Within the first 24 h of infection, the shrimp survival rate decreased sharply, accounting for the death of 70% of shrimps. All the shrimp died after 48 h, while the shrimps cultured with sterile seawater were all alive (Figure 8). The Gehan–Breslow–Wilcoxon test showed that the survival rate differed significantly between the control group and the *Vibrio*-infected group (*p* < 0.001).

## 3. Discussion

The ammonia concentration used in our experiments is based on the LC50 value, which was a concentration that kills 50% of the tested organisms. It is an important indicator of acute toxicity that was used to examine relationships between the toxicant and other abiotic or biotic variables [19]. In the present study, the LC50 of *L. vannamei* under 48 h ammonia stress was 71.68 mg/L, which may occur in aquaculture ponds. Moreover, the accumulation of feed could increase the concentration of ammonia. For example, at a feeding rate of 100 kg ha^−1^ d^−1^, ammonia concentration reached 317 mg N m^−2^ d^−1^ [20].

As an important detoxifying organ, the degree of damage to the hepatopancreas reflects the response of the organism to environmental pressure [21,22]. Here, we demonstrated that ammonia exposure led to the dilation of the hepatopancreatic lumen and tissue disorder in the shrimp *L. vannamei*. This finding is consistent with other studies [10,13,14] on ammonia-induced hepatopancreas damage in shrimp. For example, after 96 h of exposure to 45 mg/L ammonia, the hepatic tubules of *Marsupenaeus japonicus* were deformed and damaged, and increased vacuolization and epithelial cell necrosis were observed in the interstitial sinus [14]. The hepatopancreas is linked to the immune system of shrimp, and any damage to it signals oxidative stress [10,13]. As a result, reactive oxygen species (ROS) interfere with the normal physiological function of immune cells, leading to an imbalance in the homeostasis of the internal environment and ending up with the invasion of bacterial pathogens.

The rarefaction curves approached saturation, indicating that the sequencing depth was sufficient to cover the microbial diversity in the gut samples. The gut microbial diversity in shrimps decreased significantly after exposed to ammonia, suggesting that the evenness and richness of the gut microbiota are affected. This finding corroborates the results in a previous study that showed significantly decreased diversity of gut microbiota after treating *L. vannamei* with 20 mg/L ammonia for 96 h [18]. The decrease in the diversity can be attributed to the biased increase in certain genera, such as *Vibrio* and *Photobacterium*, and the loss of many other bacteria, such as *Formosa*, *Ruegeria*, and *Thalassobius*. Since a decrease in taxonomic diversity can affect the functional stability of a microbial community [23,24], the decrease in diversity can be a hallmark of the impact of excess ammonia.

*Formosa*, members of the phylum Bacteroidetes, are identified in the gut environments of several animals, such as the abalone *Haliotis gigantea* [25] and the sea cucumber *Apostichopus japonicus* [26]. In that study, *Formosa* accounted for 87.38% abundance in the hindgut of *A. japonicus* [26], suggesting important roles for host health. *Ruegeria*, belonging to the *Roseobacter* clade (or Roseobacteraceae family), are also known for their formation of biofilms [27,28] and association with marine animals [25,29]. For instance, *Ruegeria* is abundant in *H. gigantea* [25]; functionally, *Ruegeria* can produce B- vitamins, which are required by both animals and plants [29]. Moreover, *Roseobacter* clade are well known for their ability to inhibit *Vibrio* through antibiotics (e.g., tropodithietic acid) [30]. Thus, the decrease in the abundance of *Ruegeria* may partially explain the proliferation of *Vibrio*, which causes a “secondary victimization” to the shrimp.

*Vibrio* are ubiquitous in marine biofilms [31,32]; increasing evidence indicates that *Vibrio* are notorious destroyers that can break into the guts of injured marine animals. Here, we demonstrated that, ammonia exposure leads to the proliferation of *Vibrio* members affiliated with several distinct ASVs. Notably, *V. harveyi* was the most abundant *Vibrio* ASV induced by ammonia, differing from those reported in several studies on shrimp diseases [33,34,35]. For example, a seasonal juvenile vibriosis affecting *Litopenaeus stylirostris* in grow-out ponds in New Caledonia was caused by *V. penaeicida* [33], *V. parahaemolyticus* were observed in the hemolymph of moribund shrimp following disease outbreak [34], and a well-known acute hepatopancreatic necrosis disease was frequently found to be caused by *Vibrio parahaemolyticus* [35]. These results suggest the species-level specificity of *Vibrio* infection under different conditions. Members of *Photobacterium* are common causes of diseases in marine animals. Two pathogenic *Photobacterium* strains isolated from *Exopalaemon carinicauda* may lead to the death of *E. carnicauda* and *L. vannamei* [36]. In the current study, *Photobacterium* were enriched in the dead shrimps after ammonia exposure, further confirming their roles in pathogenicity.

In line with the change in taxonomic community structure, a prediction of the phenotypic and functional profiles revealed changes in the microbial metabolism after ammonia exposure. The disappearance of aerobic microbes after ammonia exposure can be explained by the proliferation of *Vibrio* species, most of which are facultative anaerobes [37,38]. These fast-growing *Vibrio* strains consume oxygen in the gut environment, resulting in the death of aerobes. Considering the functions related to pathogenicity, the relative abundance of genes encoding for MSHA, T2SS, T3SS, T6SS, toxins, and multidrug components were significantly increased after exposure to ammonia. These functions are involved in biofilm formation, host colonization, and virulence. For example, MSHA is a type of pilus that promotes bacterial adherence to host cells [39]. T3SS can manipulate and induce host cells towards cell uptake, inhibit phagocytosis, stimulate inflammation, and induce autophagy [40]. Hemolysin is an important virulence factor expressed by the *Vibrio* genus and helps in the infection process by lysing erythrocyte membranes with the release of hemoglobin [41]. Thus, these functions can also be attributed to the proliferation of *Vibrio*; further studies are required to explore the detailed functions that can explain the infection processes facilitated by ammonia exposure.

Finally, the pathogenicity of *V. harveyi* was experimentally demonstrated by isolating them and allowing them to infect healthy shrimps. In general, the bacterial cell concentration for the inoculum was 10^6^ CFU/mL, an optimal concentration for aquatic species [42]. The zero-survival rate after 48 h infection suggests a strong pathogenicity of this strain. Therefore, it can be used as a model strain for further study of the molecular basis for shrimp death during ammonia exposure. Together, this study reveals the phenotypic and functional basis for shrimp death due to ammonia exposure in terms of gut microbiota, with the loss in normal diversity, normal oxygen gradient, beneficial microbes, and pathogen proliferation. This study highlights the pathogenicity of Vibrio infections in shrimp and explains the mechanism underlying the high shrimp mortality following ammonia exposure, which shed new light on the mechanism of disease in shrimp under environmental changes.

## 4. Materials and Methods

### 4.1. Shrimp Sample Collection and Culture Condition

Healthy juvenile white shrimp (*L. vannamei*), with an average weight of 0.40 ± 0.10 g and a length of 3.78 ± 0.60 cm, were obtained from a Yantai Sanshiliwan Fisheries Technology LLC (Yantai City, Shandong Province, China). Before the experiment, shrimps were maintained in continuously aerated, running seawater (salinity 30‰, Ph 7.80 ± 0.20, and 28 ± 2 °C) under a 12:12 h light/dark (LD) cycle in a tank for 7 days. Shrimps were fed on commercial food at a frequency of four-to-six times per day. The dying ones in the culture were removed immediately. Every day, 60% of culturing seawater was replaced with fresh seawater.

### 4.2. Acute Toxicity Test of Ammonia and the Determination of 48 h Lethal Concentration (LC50)

A preliminary test was conducted to establish the ranges of lethal concentration. The toxicity period lasted for 2 days (48 h). After the acclimation for 14 days, feeding was discontinued 24 h before the commencement of the bioassay. A stock solution of ammonia was prepared by mixing NH_4_Cl (Macklin, Shanghai, China) in 1000 mg/L sterilized water. For the acute toxicity study, seven graded concentrations of NH_4_Cl solution were used in different groups: T0 (0.00 mg/L), T1 (25 mg/L), T2 (50 mg/L), T3 (75 mg/L), T4 (100 mg/L), T5 (125 mg/L), and T6 (150 mg/L). The experiments were conducted in 27 L aquaculture tanks (30 cm × 30 cm × 30 cm), each tank with 14 shrimps, and all water quality parameters were maintained in the appropriate range (salinity 30‰, pH 7.80 ± 0.20, and 28 ± 2 °C). For each concentration, three replicates were established. The mortality of *L. vannamei* was recorded every 12 h, and dead shrimp were promptly removed from the tanks. A 48 h lethal median concentration (LC50) was determined following the Karber method [43].

### 4.3. Acute Ammonia Exposure at LC50 and Sample Preparation

Shrimps were randomly picked up and cultivated in two tanks. In one tank, NH_4_Cl was added at the LC50 concentration and it was defined as the ammonia group, while in another tank, the same volume of sterilized water was added and it was defined as the control group. After exposed to acute ammonia, all the alive individuals were referred to the diseased group, while all the dead individuals were referred to the dead group. After 48 h, all the shrimps were collected, and their body length and weight were recorded. The shrimp were sterilized using 70% ethanol and dissected in a sterile environment. The dissected tissues, the hepatopancreas and gut tissues, were washed twice with 4× phosphate-buffered saline (PBS, Sangon, Shanghai, China). The whole hepatopancreas was collected for histological analysis and fixed with 4% paraformaldehyde (Biosharp, Hefei, China). The whole gut tissue was collected, frozen immediately in liquid nitrogen, and then stored at −80 °C for further analysis.

### 4.4. Histological Assay of the Hepatopancreas after Ammonia Exposure

For each group, the whole hepatopancreas from 5 shrimps were collected for histological analysis. After being fixed in 4% paraformaldehyde for 24 h, the hepatopancreas tissues were dehydrated using a gradient solution of methanol (25%, 50%, and 75%) with each step lasting 30 min and embedded in paraffin using embedding equipment (Leica Biosystems HistoCore Arcadia, Leica Biosystems, Wetzlar, Germany). The sections were continuously sliced with a fully automated rotary microtome HistoCore AUTOCUT (Leica Biosystems, Wetzlar, Germany) to 4~5 μm thicknesses, spread in a water bath at 40 °C, mounted on glass slides (three to four serial sections per slide), and dried at 37 °C overnight. The wax was removed from the hepatopancreas tissue sections using xylene and ethanol, rehydrated in a decreasing ethanol gradient (100%, 95%, 80%, 70%, 50%, and 30%; 2 min for each step), and stained with hematoxylin–eosin (H&E) (Njjcbio, Nanjing, China). All the stained slides were dehydrated in ethanol and xylene (Macklin, Shanghai, China) for 5 min each, sealed with neutral gum (Solarbio, Beijing, China), and then observed under a BX43 manual light microscope (Olympus, Tokyo, Japan).

### 4.5. DNA Extraction and Sequencing of 16S rRNA Gene Amplicon

For each group, the whole gut tissue from 17 shrimps was collected to extract the genomic DNA. The total DNA of the gut microbiome was extracted using the TIANamp Genomic DNA Kit (Tiangen, Beijing, China), according to the manufacturer’s instructions. Amplification of the V3-V4 region of 16S rRNA genes from the gut microbial communities was performed using primers with the barcodes 341F: CCTAYGGGRBGCASCAG and 806R: GGACTACNNGGGTATCTAAT [44]. The PCR reaction mixture contained 15 μL of Phusion^®^ High-Fidelity PCR Master Mix (New England Biolabs, Ipswich, MA, USA), 0.2 μm of F/R primers, and 10 ng of a DNA template to a final volume of 50 µL with distilled H_2_O. The PCR program was 98 °C for 1 min, followed by 30 cycles of amplification (98 °C for 10 s, 50 °C for 30 s, and 72 °C for 30 s), and 72 °C for 5 min for the final extension. The PCR products were analyzed by 2% agarose gel electrophoresis.

The PCR products were purified using a Qiagen Gel Extraction Kit (Qiagen, Hilden, Germany) following the manufacturer’s instructions. DNA libraries were constructed, and the quality was assessed on a Qubit@2.0 Fluorometer (Thermo Scientific, Waltham, MA, USA) and an Agilent Bioanalyzer 2100 system. Then, DNA libraries were sequenced on an Illumina NovaSeq platform to generate 250 bp paired-end reads. Each sample was sequenced to a depth of 500,000 tags; the sequencing information of this study is presented in Supplementary Table S1.

### 4.6. Sequencing Analysis of 16S rRNA Gene Amplicon

The 16S rRNA gene paired-end raw reads were assigned to samples according to unique barcode sequences and then merged into raw tags using FLASH v1.2.7 [45]. The merged reads with low-quality reads with ambiguous bases or truncated at any site with more than three subsequent bases were filtered, and any with a Phred quality score < 20 were removed by Trimmomatic v0.39 [46]. Chimera and noises were removed using VSEARCH v2.8.1 [47] and QIIME2 v2020.11 [48] to generate clean reads. A total of 39,086 clean reads were randomly selected from each sample for normalizing the uneven sequencing depth. Classification of the amplicon sequence variants (ASVs) was performed de novo with 100% sequence similarity, and the representative sequences were recovered using DADA2 [49]. The abundance of ASVs was calculated from the normalized clean reads mapping to the representative sequences. The representative sequence of ASVs was taxonomically classified according to the SILVA database in Parallel-META 3 v3.4.4 [50]. Rarefaction curves and alpha diversity (Observed ASVs, Chao_1, ACE, and Shannon_n) were analyzed using the vegan, picante, ggplot2, doBy, and ggalt packages in R program [51,52]. The principal component analysis (PCoA) among different groups was performed based on Jaccard distances using vegan, ggplot2 packages in R program v4.1.1 [52,53].

The gut microbial genera with significant changes among different groups were explored based on their linear discriminant analysis (LDA) effect sizes (LEfSes) [54] and further analyzed with non-parametric factorial Kruskal–Wallis (KW) sum-rank tests. A violin plot was generated using the ggplot2 package in the R program, depicting the distribution and variability in the log10 relative abundance of *Vibrio* among various individuals across different groups [55] and statistically analyzed with a two-tailed Student’s *t*-test. The microbial phenotype categories (aerobic, anaerobic, facultatively anaerobic, and potentially pathogenic phenotypes) were predicted using BugBase [56] and further assessed with a two-tailed Student’s t-test. The functional genes of the gut microbial community according to the abundance and composition of the ASVs were analyzed through the Phylogenetic Investigation of Communities by Reconstruction of Unobserved States (PICRUSt2,v2.4.2) [57] and further examined by one-way analysis of variance (ANOVA) in the Statistical Analysis of Metagenomic Profiles v2.1.3 [58].

### 4.7. Bacterial Isolation, Identification, and the Phylogenetic Relationships

For each tank, the gut tissues of the remaining shrimps were merged together for strain isolation. Bacteria were isolated from the gut of *L. vannamei* after exposure to ammonia, especially the dead shrimp. The fresh gut tissue was ground thoroughly in sterile conditions after washing twice with 4 × PBS. After diluting 100 times, a 100 µL aliquot of ground gut fluid was spread on Marine Broth 2216E plates (HOPEBIO, Qingdao, China) and incubated at 28 °C for 24 h. The bacterial colonies with consistent morphologies were passaged onto a fresh culture medium to obtain pure cultures. The isolates were purified by 7–8 generations of continuous cultivation and cultured, and DNA was extracted for further identification. For Sanger sequencing (Sangon, Shanghai, China), PCR products were amplified with the 16S rRNA genes primers 27F (5′-AGAGTTTGATCCTGGCTCAG-3′) and 1492R (5′-GGTTACCTTGTTACGACTT-3′). The 20 μL PCR reaction mixture contained 10 μL of the 2× Q5 Master Mix (NEB, Ipswich, USA), 0.5 μL of F/R primers, 1.0 μL of a DNA template, and 8.0 μL of distilled H_2_O. The PCR program was performed at 95 °C for 1 min, followed by 30 cycles of amplification (95 °C for 10 s, 52 °C for 30 s, and 72 °C for 2 min), and the final extension at 72 °C for 5 min. The PCR products were analyzed by 2% agarose gel electrophoresis. The taxonomy of 16S rRNA gene sequences was further classified by an RDP classifier [59]. The phylogenetic tree of the isolate and its relatives was constructed using a 16S rRNA gene sequence. All the sequences were aligned using ClustalW, and a maximum composite likelihood tree was constructed in MEGA v10.2.6 [60]. Bootstrap values were calculated based on 1000 replicates.

### 4.8. The Pathogenic Bacteria Infection of Shrimp

To examine the pathogenic of bacteria, the shrimp were exposed to bacterial infection. All the shrimps were kept in laboratory conditions for three days before the pathogenic challenge. Bacteria were cultured in a Marine Broth 2216E medium at 28 °C at 180 rpm and collected in the logarithmic phase (OD600 = 0.6). This was centrifuged at 1500× *g* for 10 min, and the bacteria were washed twice in sterile seawater. Shrimps were randomly cultured in six tanks with 50 individuals for each tank. To three tanks, 1 × 10^6^ CFU/mL of bacterial cells were added, and an equal volume of sterile seawater was added in the other three tanks. The shrimp were monitored at regular intervals until they were stable and no further mortality was observed. The mortality rate of shrimp was calculated based on the Kaplan–Meier method in GraphPad Prism v8.0.2 and statistically analyzed with a Gehan–Breslow–Wilcoxon test [61].

## Data Availability

Raw sequencing data of 16S sequences can be found on the NCBI sequence read archive under Bioproject_ID: PRJNA1048123. Additional data will be made available on request.

## Supplementary Materials

The following supporting information can be downloaded at https://www.mdpi.com/article/10.3390/ijms25052614/s1.
