# Supplementary material for: Acute Ammonia Causes Pathogenic Dysbiosis of Shrimp Gut Biofilms
Source: Int J Mol Sci. 2024 Feb 23;25(5):2614. doi: 10.3390/ijms25052614 (PMC10932075; doi:10.3390/ijms25052614)
Supplement: Supplementary file 1 [file ijms-25-02614-s001.zip › ijms-2853043-supplementary.pdf]

Acute Ammonia Causes Pathogenic Dysbiosis of Shrimp Gut Biofilms

Ning Gao [1,2,3], Yi Shu [1,2,3], Yongming Wang [1,2,3], Meng Sun [4], Zhongcheng Wei [3], Chenxi Song [3], Weipeng Zhang [4], Yue Sun [2,3], Xiaoli Hu [3], Zhenmin Bao [1,2,3,*] and Wei Ding [3,*]

1 Southern Marine Science and Engineer Guangdong Laboratory, Guangzhou 511458, China; ninggao1005@163.com (N.G.); 15254135903@163.com (Y.S.); wymsyp12@163.com (Y.W.)

2 Key Laboratory of Tropical Aquatic Germplasm of Hainan Province, Sanya Oceanographic Institution, Ocean University of China, Sanya 572000, China; sunyue@ouc.edu.cn

3 MOE Key Laboratory of Marine Genetics and Breeding, Ocean University of China, Qingdao 266003, China; weizhongcheng@stu.ouc.edu.cn (Z.W.); 13397712090@163.com (C.S.)

4 Institute of Evolution & Marine Biodiversity, Ocean University of China, Qingdao 266003, China; sunmeng950103@163.com (M.S.); zhangweipeng@ouc.edu.cn (W.Z.)

* Correspondence: zmbao@ouc.edu.cn (Z.B.); dingwei@ouc.edu.cn (W.D.)

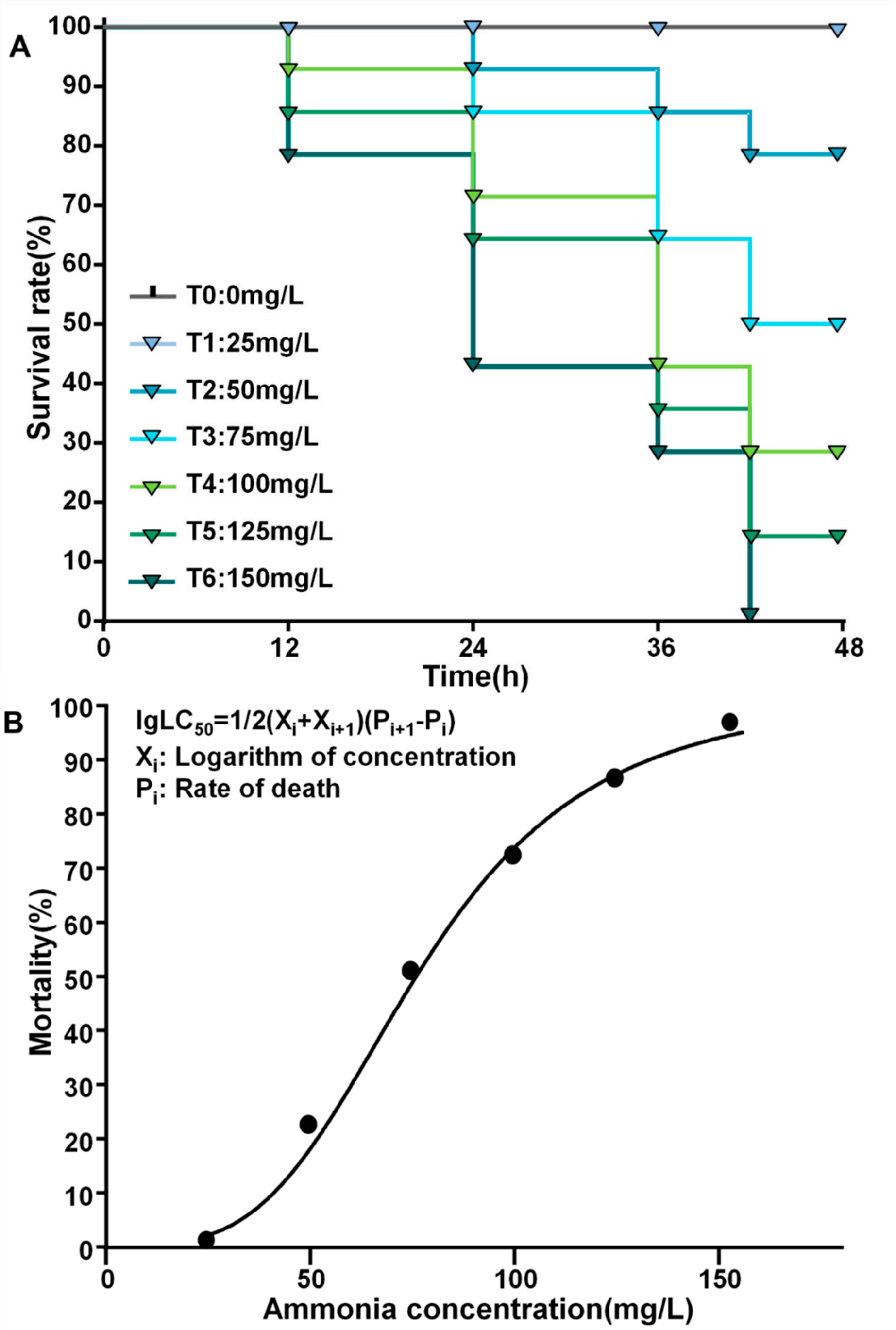

Figure S1 The effects of acute ammonia exposure on the survival of *Litopenaeus vanname.* (A) The 48 hours survival rate of shrimp *L. vannamei* after exposed to various ammonia (NH_4^+) concentrations. (B) The dose-dependent mortality of *L. vannamei* after exposed to various ammonia (NH_4^+) concentrations.

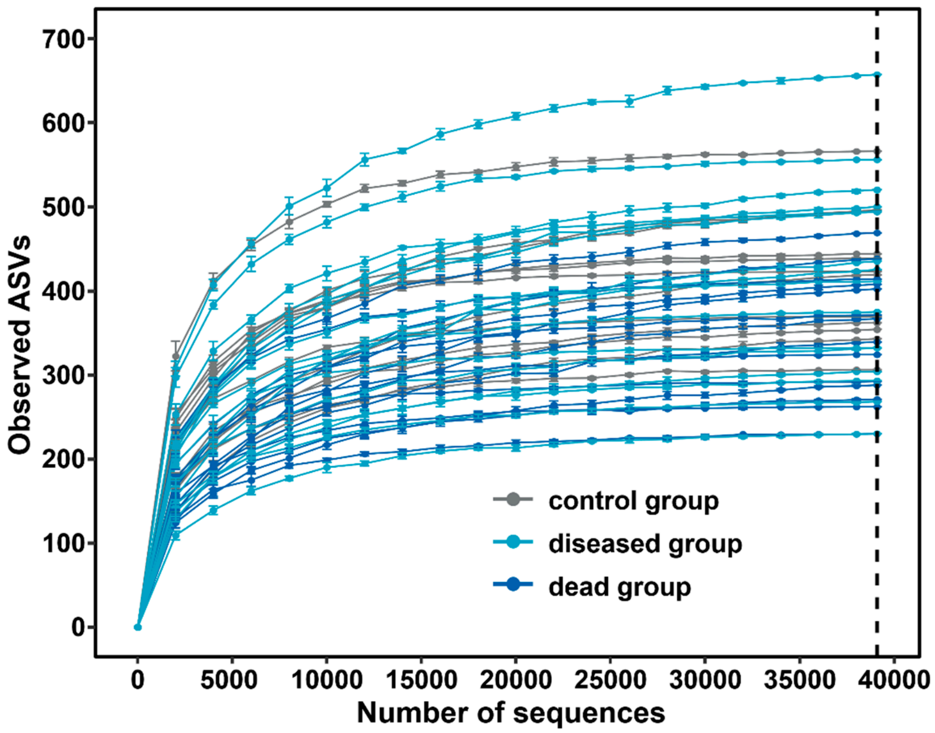

Figure S2 Rarefaction curves of 16S rRNA sequencing depth and detected amplicon sequence variants (ASVs). The calculations were performed for 5 permutations after normalizing all the samples to nearly 40,000 reads.

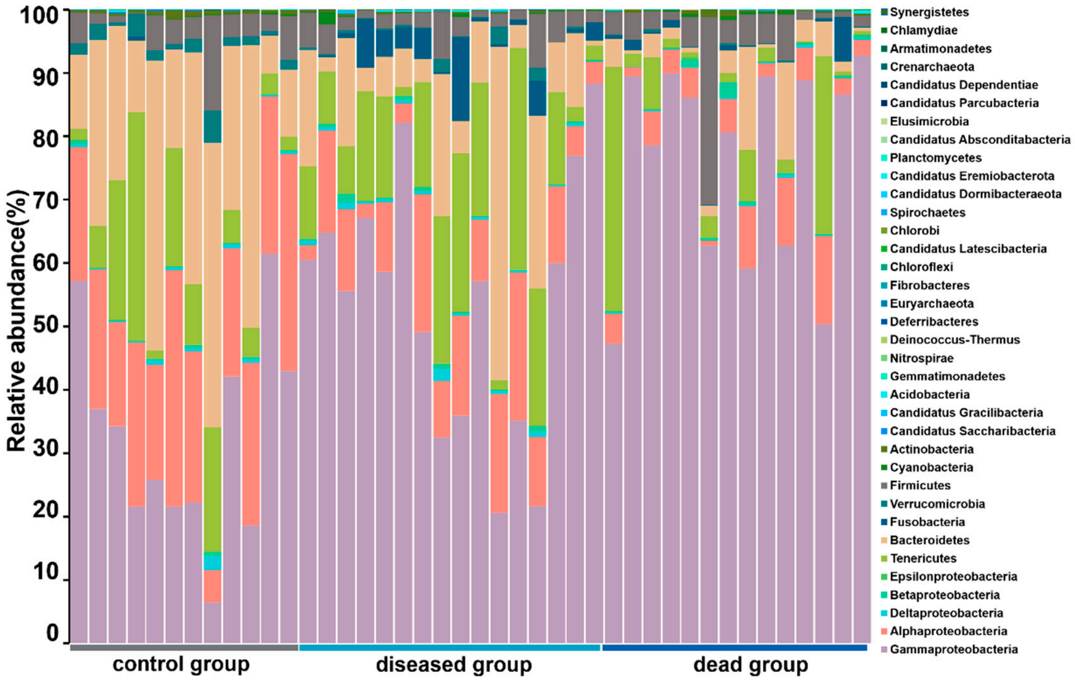

Figure S3 Taxonomic structure of the gut microbiota at the phylum level. Proteobacteria were further classified to the class level.

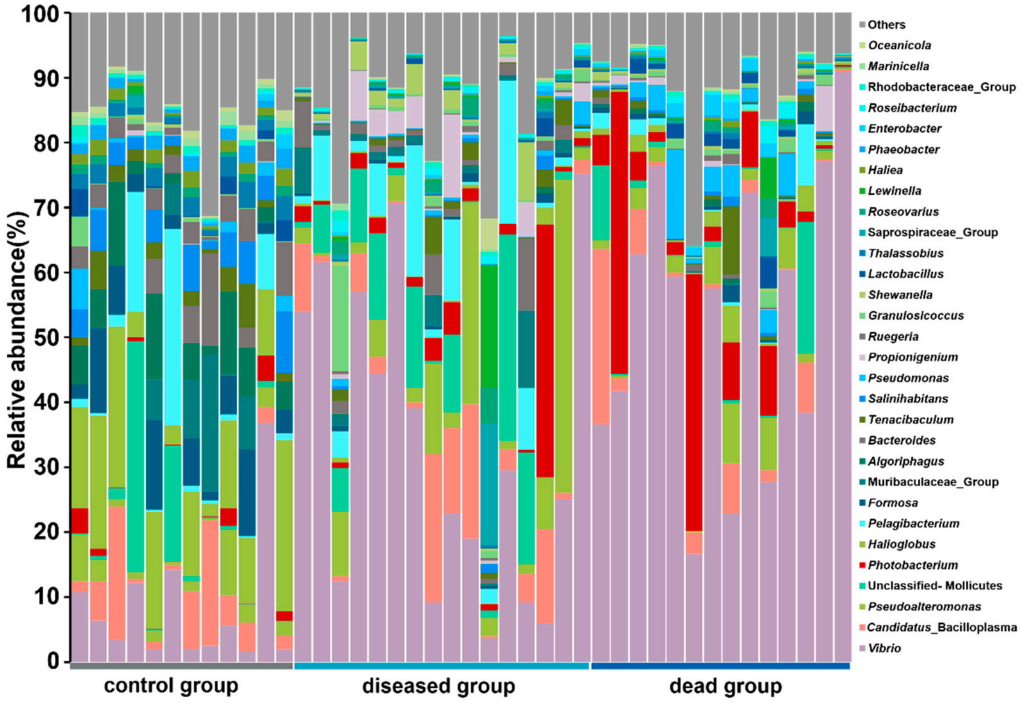

Figure S4 Taxonomic structure of the gut microbiota at the genus level. The top 30 genera ranked by the relative abundance among the 42 samples are shown, while other genera are summarized as 'Others'.

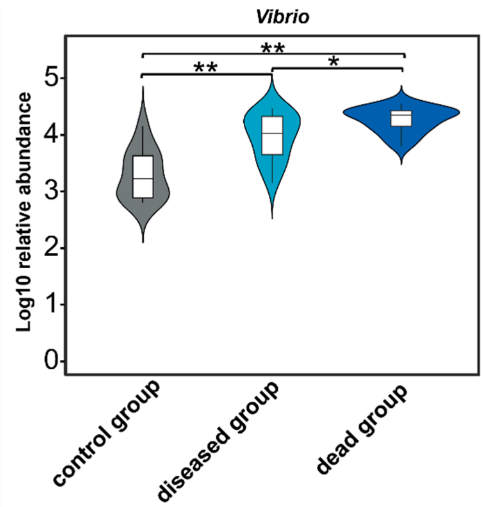

Figure S5 The violin plots to show the relative abundances (log10) of *Vibrio* strains in three groups. Two-tailed student t-test was performed to assess the statistical variation of *Vibrio* between each pair of groups. *$P < 0.05$, **$P < 0.01$.

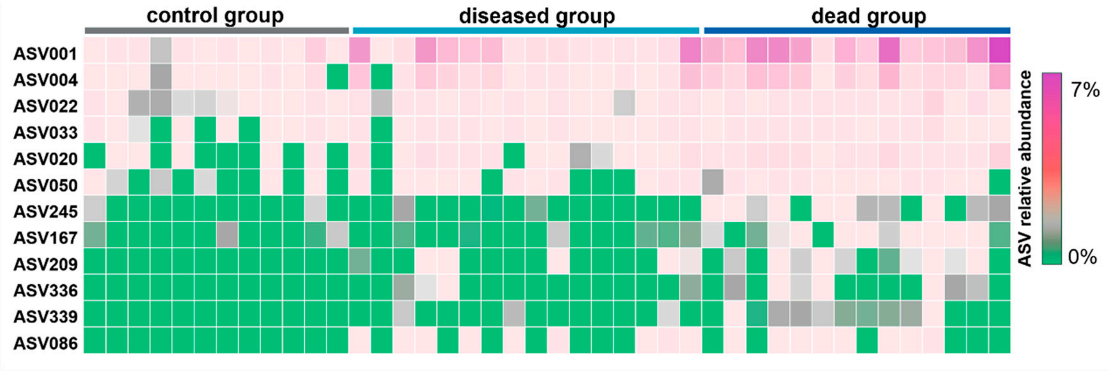

Figure S6 The heatmap visualization of 12 significantly changed ASVs of *Vibrio* across different groups. One-way ANOVA was used to identify the significantly changed ASVs across different groups($P<0.05$).

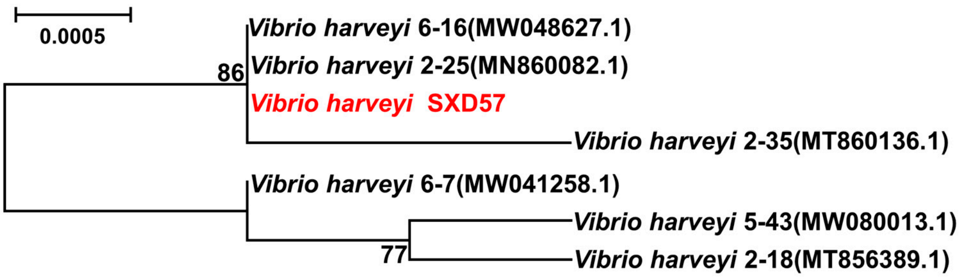

Figure S7 Phylogenetic relationships between SXD57 and other related bacteria based on 16S rRNA gene sequences. The phylogenetic tree was generated using the maximum-likelihood method with 1000 bootstraps.

Table S1 Information of 16S rRNA gene amplicon sequences.

Sample Name	Data size(bp)	Total number of clean reads	Average of read length(bp)	Maximum of read length (bp)	Minimum of read length (bp)
control-1	19,027,986	51917	423	430	103
control-2	30,306,054	82411	421	430	50
control-3	32,136,671	85914	423	430	54
control-4	30,502,890	82096	424	430	53
control-5	27,475,456	79986	421	430	14
control-6	30,979,534	82033	423	430	64
control-7	17,694,619	50098	420	430	50
control-8	25,438,341	77019	421	430	88
control-9	31,440,998	87921	422	430	227
control-10	26,326,459	79744	419	430	262
control-11	32,199,134	84733	423	430	205
control-12	20,699,770	58483	419	430	54
diseased-1	26,204,315	69489	426	430	54
diseased-2	33,228,679	81606	425	430	15
diseased-3	34,301,794	90808	424	430	58
diseased-4	32,388,503	82223	426	430	261
diseased-5	31,062,221	77632	426	430	52
diseased-6	31,871,054	79289	426	430	53
diseased-7	34,788,859	85700	425	430	15
diseased-8	23,587,028	64713	423	430	52
diseased-9	33,896,782	84445	423	430	241
diseased-10	28,522,776	73097	425	430	99

diseased-11	27,363,796	84353	421	430	15
diseased-12	29,261,974	72219	426	430	104
diseased-13	29,991,024	84620	423	430	123
diseased-14	33,405,696	86601	425	430	119
diseased-15	19,681,009	50741	426	430	223
diseased-16	34,154,159	84023	427	430	223
dead-1	35,342,960	87011	426	430	111
dead-2	22,097,724	55929	427	430	243
dead-3	35,525,898	89943	427	430	228
dead-4	31,902,053	79517	428	430	239
dead-5	25,503,132	63996	426	430	53
dead-6	34,672,111	87996	422	430	15
dead-7	20,697,495	53747	427	430	292
dead-8	32,447,512	86202	425	430	64
dead-9	32,994,776	82524	428	430	224
dead-10	20,949,579	57916	425	430	262
dead-11	32,427,027	80717	427	430	110
dead-12	35,539,170	89253	426	430	51
dead-13	34,328,415	83585	426	430	52
dead-14	31,912,806	76631	428	430	52
